# Antibiotics for Fever Among Children: Findings From the Surveillance for Enteric Fever in India Cohorts

**DOI:** 10.1093/infdis/jiab115

**Published:** 2021-11-23

**Authors:** Arun S Karthikeyan, Manikandan Srinivasan, Suman Kanungo, Bireshwar Sinha, Ankita Shrivastava, Karthikeyan Ramanujam, Santhosh Kumar Ganesan, Sathyapriya Subramaniam, Kulandaipalayam Natarajan Sindhu, Swathi Krishna, Prasanna Samuel, Winsley Rose, Venkata Raghava Mohan, Balaji Veeraraghavan, Temsunaro Rongsen-Chandola, Shanta Dutta, Ashish Bavdekar, Jacob John, Gagandeep Kang

**Affiliations:** 1 Christian Medical College, Vellore, India; 2 National Institute for Cholera and Enteric Diseases, Indian Council of Medical Research, Kolkata, India; 3 Centre for Health Research and Development–Society for Applied Studies, New Delhi, India; 4 KEM Hospital and Research Centre, Pune, India

**Keywords:** antibiotic treatment, acute febrile illness, pediatric, cohort, India, SEFI

## Abstract

**Background:**

Acute febrile illness in children is frequently treated with antibiotics. However, the inappropriate use of antibiotics has led to the emergence of multidrug-resistant pathogens.

**Methods:**

We measured use of antibiotics for fever in 4 pediatric cohorts that were part of the Surveillance for Enteric Fever in India (SEFI) network. In this network, 24 062 children were followed up weekly, capturing information on fever and other morbidity between October 2017 and December 2019.

**Results:**

An antibiotic was given in 27 183 of the 76 027 (35.8%) episodes of fever. The incidence of fever-related antibiotic use was 58.0 (95% confidence interval [CI], 57.2–58.6) per 100 child-years. The median time to initiation of antibiotics was 4 days, and in 65% of those who received an antibiotic it was initiated by the second day. Antibiotics were continued for <3 days in 24% of the episodes. Higher temperature, younger age, male sex, joint family, higher education, internet access, and availability of personal conveyance were associated with antibiotic treatment for fever.

**Conclusions:**

In developing countries where antibiotic use is not regulated, broad-spectrum antibiotics are initiated early, and often inappropriately, in febrile illness. Frequent and inappropriate use of antibiotics may increase risk of antimicrobial resistance.

Acute febrile illness with or without other symptoms is common in children [1]. While antibiotics are essential for the treatment of bacterial infections [2], their indiscriminate use is not only therapeutically ineffective, but also facilitates antimicrobial resistance [3, 4]. This is a particular problem in low- and middle-income countries (LMICs), given their higher burden of infectious diseases. Furthermore, lack of access to healthcare and rapid, accurate diagnostics contribute to the overuse of antibiotics, especially when delay can result in complications [3]. Recently, the mean number of antibiotic prescriptions in children under 5 years of age in LMICs was estimated at 24.5 episodes per child [5]. Global antibiotic consumption was estimated as 34.8 billion defined daily doses in 2015 [6], reflecting an average of 5 days of antibiotic use per person. The low per-person consumption of antibiotics in the LMICs, combined with high resistance rates, suggests a complexity of demographic and socioeconomic factors that determine inappropriate antibiotic use [7].

The Surveillance for Enteric Fever in India (SEFI) network established pediatric cohorts in 4 locations in India to study the burden of culture-confirmed enteric fever [8]. Each cohort comprised around 6000 children <15 years of age who were followed up for 2 years. We documented episodes of fever and the incidence of antibiotic usage for fever.

This analysis estimates the frequency and patterns of antibiotic use in children for febrile illness, including incidence of fever-related antibiotic use and the choice, timing, and duration of antibiotics. In addition, we examined the demographic and socioeconomic factors associated with antibiotic usage for fever.

## METHODS

The study protocol was approved by the Institutional Review Board of the Christian Medical College and each participating institute. Written informed consent was obtained from parents of participating children and assent from age-eligible children.

### Study Sites

In the SEFI network, 4 cohorts of children, between 6 months and 15 years of age, were recruited and followed up, in 3 urban (Delhi, Kolkata, and Vellore) settings and a rural area on the outskirts of Pune. In Delhi, the study was conducted in 2 contiguous blocks of Sangam Vihar, a low-income urban resettlement neighborhood in the south of the city. In Kolkata, the study area consists of wards 58 and 59, a predominantly impoverished slum area located in the eastern part of the city. In Vellore, the study area comprised the contiguous semi-urban settlements of Chinallapuram, Kaspa, Ramnaickanpalayam, and Vasanthapuram. In Pune, the study area, located 30 km from the city, was a rural, agrarian settlement. The urban sites had a slightly higher literacy rate than the rural site (Delhi 89.4%, Kolkata 88.1%, and Vellore 89.0% vs Pune 79.4%) [9]. The study sites are described in detail elsewhere [8]. The study enrolled approximately 6000 children at each location. Follow-up of children in this cohort continued until completion of 2 years from enrollment, attainment of 15 years of age, or premature loss to follow-up, whichever occurred earlier.

### Fever Surveillance and Antibiotic Data Collection

A weekly interview of the caregiver of the participant was conducted by a trained field research assistant to capture morbidity and hospitalizations. For missed visits, recall for up to 14 days was considered valid and any longer period was interval-censored. Those identified with fever were contacted daily, until resolution of the fever. During these visits, information about daily highest temperature, visits to any healthcare facility, and administration of medicines like antipyretics and antibiotics was collected. The peak temperature is the maximum documented temperature from the fever diary card on a calendar day. Fever of notable severity (FNS) was evaluated by a study physician and a blood culture was offered to identify the etiology. The study personnel documented the antibiotics taken by the study children, and the study physician confirmed the diagnosis and antibiotic details. A study physician, blinded to the actual antibiotic use, reviewed the symptoms, investigation results, and clinical diagnosis documented by the treating physician and classified FNS episodes into probable, possible, and unlikely bacterial etiology.

For the purposes of the study, the following terms were used and defined. Any subjective report of fever, irrespective of documented temperature, was considered a fever episode. Resolution of fever episode was defined as 3 consecutive afebrile days. FNS was defined as an episode of fever with at least 3 consecutive days of fever. Antibiotics related to a fever episode was defined as an antibiotic started between the onset of fever and 1 day beyond the last febrile day. Multiple antibiotics related to a fever episode was defined as >1 class of antibiotics given during a single fever episode. Inappropriate antibiotics were defined based on 3 characteristics—timing of initiation, duration of antibiotics, and indication for antibiotic. Early initiation of antibiotics related to fever episode was defined as an antibiotic started before 3 days of fever was completed. Short duration antibiotic usage for fever episode was defined as antibiotic usage for a period of <3 days during an episode of fever. Antibiotic usage for inappropriate indication was defined as an antibiotic used for FNS with an unlikely bacterial etiology.

### Statistical Analysis

The incidence of episodes of fever and antibiotic use was estimated as the rate of events over person-time of observation. We assumed and allowed for repeated occurrence of events and included valid periods of observation in person-time calculation. To determine the most common combination of antibiotics used in the study population, “UpsetR” graph was used [10]. The median time to initiation of antibiotics was determined using the Kaplan–Meier survival analysis. To determine the sociodemographic and other risk factors associated with antibiotic usage for episodes of fever, generalized estimating equations were used. Statistical analyses were performed using Stata software version 14.2 (Stata Corp), and graphics were generated using R (version 3.6.1).

## RESULTS

### Acute Febrile Illness

**Figure 1.**
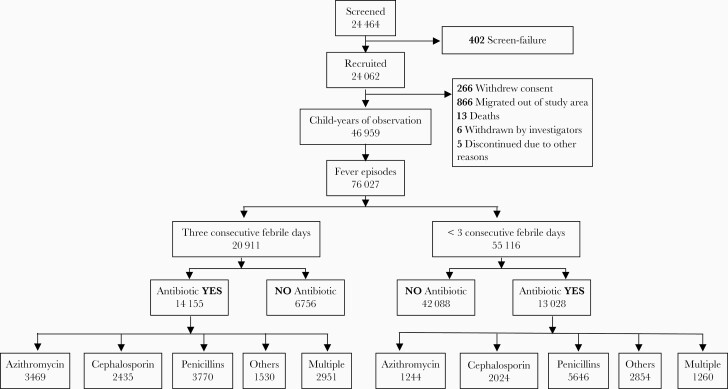
Flow diagram of recruitment and antibiotic treatment for acute febrile illness among the pediatric cohorts established as a part of the Surveillance for Enteric Fever in India (SEFI) network.

The combined cohort of 24 062 children from the 4 sites contributed to 46 959.3 child-years of observation (CYO) until 31 December 2019 (Figure 1). There were 76 027 episodes of fever, with incidence of fever estimated as 1.62 (95% confidence interval [CI], 1.61–1.63) episodes per CYO among children between 6 months and 15 years of age. The incidence of fever was highest in the age group 6 months to 4 years (2.45 [95% CI, 2.43–2.48] episodes per CYO), followed by children aged 5–9 years (1.53 [95% CI, 1.51–1.55] episodes per CYO) and 10–14 years (1.14 [95% CI, 1.13–1.16] episodes per CYO). The mean duration of fever was 2.41 days (standard deviation, 1.7 days). There were 20 911 (27.5%) episodes of FNS, and 812 (1.1%) episodes of fever required hospitalization (Table 1).

**Table 1.** Summary of the Acute Febrile Illness Among the 4 Pediatric Cohorts in India Established as Part of the Surveillance for Enteric Fever in India (SEFI) Network

Characteristic	Site				
	Delhi	Kolkata	Vellore	Pune	Overall
Fever episodes, No.	14 439	17 741	23 548	20 299	76 027
Age group					
6 mo–4 y	5612 (38.9%)	6175 (34.8%)	9505 (40.4%)	7503 (37.0%)	28 795 (37.9%)
5–9 y	5412 (37.5%)	6071 (34.2%)	8710 (37.0%)	7441 (36.7%)	27 634 (36.3%)
10–14 y	3415 (23.7%)	5495 (31.0%)	5333 (22.7%)	5355 (26.4%)	19 598 (25.8%)
Duration of fever					
Mean (SD), d	2.72 (2.2)	2.25 (1.6)	2.46 (1.8)	2.26 (1.4)	2.41 (1.7)
1 d	4724 (32.7%)	6560 (37.0%)	9080 (38.6%)	6793 (33.5%)	27 157 (35.7%)
2 d	4294 (29.7%)	6462 (36.4%)	6197 (26.3%)	7181 (35.4%)	24 134 (31.7%)
≥3 d	5421 (37.5%)	4719 (26.6%)	8271 (35.1%)	6325 (31.2%)	24 736 (32.5%)
Fever of notable severity	4198 (29.1%)	4285 (24.2%)	6878 (29.2%)	5550 (27.3%)	20 911 (27.5%)
Hospitalization	101 (0.7%)	91 (0.5%)	309 (1.3%)	311 (1.5%)	812 (1.1%)
Agreement between provisional and final diagnosis	68.5%	86.7%	75.1%	99.5%	82.2%
Antibiotic usage	5270 (36.5%)	5164 (29.1%)	6377 (27.1%)	10 372 (51.1%)	27 183 (35.8%)
Multiple antibiotics	981 (18.6%)	779 (15.1%)	850 (13.3%)	1601 (15.4%)	4211 (15.5%)
Incidence of antibiotics^a^					
At least 1	46.7 (45.5–48.0)	44.4 (43.2–45.6)	55.4 (54.0–56.7)	82.8 (81.2–84.4)	57.9 (57.2–58.6)
Multiple	8.7 (8.2–9.3)	6.7 (6.2–7.2)	7.4 (6.9–7.9)	12.8 (12.2–13.4)	9.0 (8.7–9.2)

Data are presented as No. (%) unless otherwise indicated.

Abbreviation: SD, standard deviation.

^a^Episodes per 100 child-years of observation (95% confidence interval).

### Incidence of Antibiotic Usage Related to Fever

Of the 76 027 episodes of fever, 27 183 (35.8%) episodes received antibiotics. The incidence of antibiotic usage related to fever episodes was 57.9 (95% CI, 57.2–58.6) episodes per 100 CYO among children between 6 months to 15 years of age. Again, the incidence was highest among children in the youngest age group (95.1 [95% CI, 93.4–96.9] episodes per 100 CYO) and lowest in the oldest age group (35.7 [95% CI, 34.8–36.6] episodes per 100 CYO). The incidence of multiple antibiotics being used for fever was 9.0 (95% CI, 8.7–9.3) episodes per 100 CYO (Table 1 and Supplementary Table 2).

### Choice of Antibiotics

**Figure 2.**
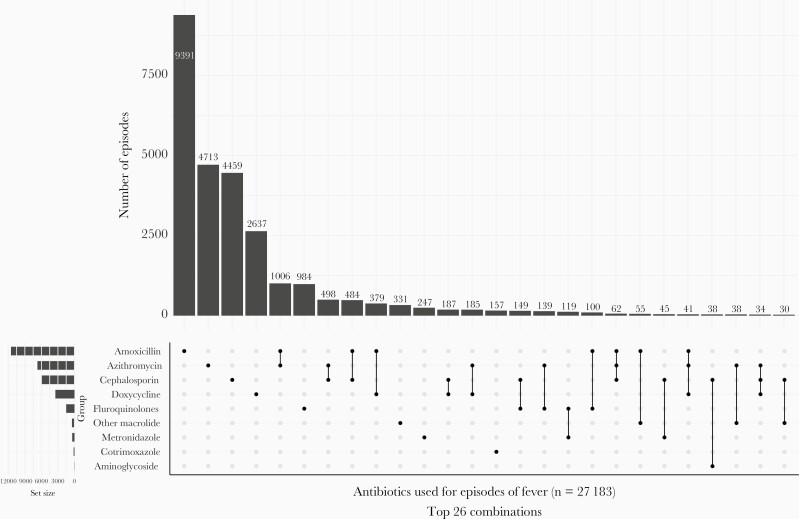
Combination of antibiotics used for acute febrile illness among the 4 cohorts established as a part of the Surveillance for Enteric Fever in India (SEFI) Network.

Amoxicillin (25.1 [95% CI, 24.6–25.6] episodes per 100 CYO), followed by azithromycin (14.8 [95% CI, 14.4–15.1] episodes per 100 CYO) were the most frequently used antibiotics for fever (Supplementary Table 3). However, 4211 (16%) febrile episodes received multiple antibiotics (Figure 1). The most common combination of antibiotics for the same episode of fever were azithromycin and amoxicillin, used for treating 1006 episodes (Figure 2). Finally, a small subset (<1%) of fever episodes received unknown substances from informal sources (including traditional healers, nonqualified practitioners, neighbors, and relatives). These drugs did not have labels and the study team was unable to ascertain if they were antibiotics.

### Timing and Duration of Antibiotics

**Figure 3.**
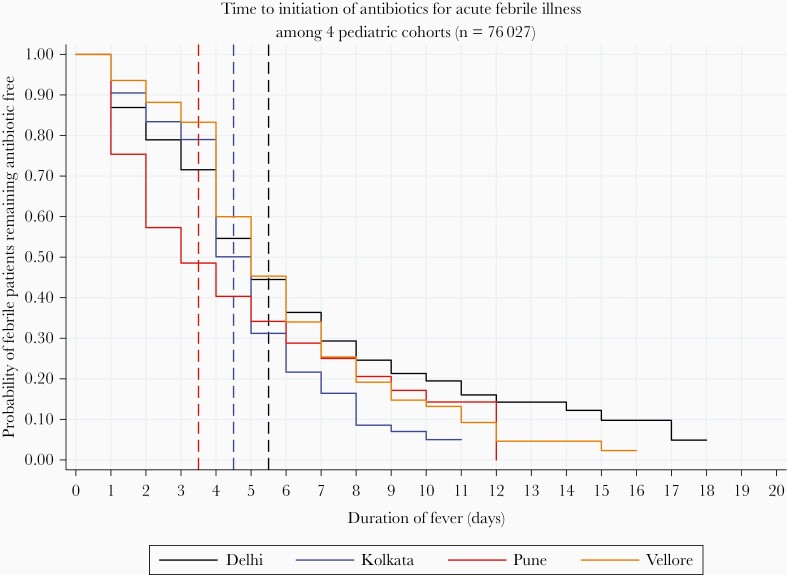
Probability of febrile patients remaining antibiotic free by sites among pediatric cohorts established as a part of the Surveillance for Enteric Fever in India (SEFI) network.

The median time to initiation of antibiotic from the onset of fever was estimated at 4 days (Supplementary Figure 1), ranging from the third day at the Pune site to the fifth day for the Delhi and Vellore sites (Figure 3). Among those fever episodes that received an antibiotic, 64% of the fever episodes had an early initiation of antibiotics, ranging from 44% in Vellore to 84% in Pune. In all the episodes of fever which were treated with antibiotics, treatment was for <3 days in 24.3% of the cases. This ranged between from 8% in Kolkata to 39% in Delhi (Table 2).

**Table 2.** Appropriateness of Antibiotic Usage for Acute Febrile Illness Among the 4 Pediatric Cohorts Established as Part of the Surveillance for Enteric Fever in India (SEFI) Network

Indicators	Site				
	Delhi	Kolkata	Vellore	Pune	Overall
Time to initiation, d, mean (SD)	2.60 (1.7)	2.59 (1.5)	3.06 (1.7)	1.78 (1.0)	2.39 (1.5)
Antibiotic course duration, d, mean (SD)	3.04 (1.6)	4.31 (1.5)	3.29 (1.8)	3.34 (1.2)	3.45 (1.6)
Early initiation (in first 2 d)	57.7%	57.0%	43.7%	83.6%	64.2%
Short-duration antibiotic (<3 d)	39.3%	8.2%	26.5%	22.7%	24.3%
Antibiotic for unlikely bacterial infection (provisional diagnosis)	67.7%	59.1%	55.5%	83.7%	65.4%
Antibiotic for unlikely bacterial infection (final diagnosis)	95.8%	58.3%	71.6%	83.2%	75.9%

Abbreviation: SD, standard deviation.

Out of 12 683 episodes of FNS which had a blood culture performed, 2.8% (n = 360) had a culture-confirmed bacterial etiology. In other words, 12 323 episodes (97.2%) of FNS in which blood culture was performed did not have a culture-confirmed bacterial etiology. The final diagnosis was available for 17 617 episodes, of which acute undifferentiated febrile illness (46.4%), upper respiratory tract infection (31%), and lower respiratory tract infection (11%) were the 3 most common diagnoses (Supplementary Table 1).

We observed 82% agreement between the provisional and final diagnosis among FNS in terms of the bacterial etiology. Based on the provisional diagnosis, the antibiotic usage for an inappropriate indication was 65.4%, varying between 55.5% in Vellore and 83.7% in Pune.

### Risk Factors of Antibiotic Usage for Acute Febrile Illness

Severe illness, indicated by a higher temperature (odds ratio [OR], 1.35 [95% CI, 1.30–1.40]), longer duration of fever (OR, 2.01 [95% CI, 1.98–2.04]), and episodes requiring hospitalization (OR, 3.61 [95% CI, 2.86–4.55]), was associated with an increased risk of receiving an antibiotic (Table 3). Younger age groups had a higher risk of receiving an antibiotic as compared to children >10 years of age (OR, 1.34 [95% CI, 1.27–1.41]) for children under 5 and 1.16 [95%CI, 1.11–1.22] for those aged 5–9 years) (Table 3). Among the socioeconomic factors, those with postsecondary education (OR, 1.07 [95% CI, 1.02–1.12]), internet access (OR, 1.07 [95% CI, 1.01–1.13]), and availability of personal conveyance (OR, 1.09 [95% CI, 1.04–1.14]) at the household level reported a higher odds of receiving antibiotics for acute febrile illness.

**Table 3.** Generalized Estimating Equation Model for the Risk of Antibiotic Usage for Febrile Illness Among the 4 Pediatric Cohorts Established as Part of the Surveillance for Enteric Fever in India (SEFI) Network

Variable	Unadjusted		Adjusted	
	OR (95% CI)	*P* Value	OR (95% CI)	*P* Value
Sites				
Delhi	1.53 (1.45–1.60)	<.001	1.55 (1.46–1.65)	<.001
Kolkata	1.08 (1.03–1.13)	.003	1.44 (1.37–1.54)	<.001
Pune	2.84 (2.72–2.98)	<.001	4.21 (3.97–4.47)	<.001
Vellore	1		1	
Social factors				
Postsecondary education	1.43 (1.38–1.48)	<.001	1.07 (1.02–1.12)	.003
Internet accessibility	1.38 (1.31–1.44)	<.001	1.07 (1.01–1.13)	.019
Conveyance (motor vehicle)	1.37 (1.32–1.41)	<.001	1.09 (1.04–1.14)	.001
Nuclear family	0.93 (.90–.97)	<.001	0.90 (.86–.94)	<.001
Monthly income >10 000 INR	1.58 (1.52–1.63)	<.001	1.04 (1.00–1.09)	.073
Individual factors				
Female sex	0.93 (.90–.96)	<.001	0.94 (.90–.97)	.001
Age category				
6 mo–4 y	1.37 (1.31–1.43)	<.001	1.34 (1.27–1.41)	<.001
5–9 y	1.20 (1.15–1.26)	<.001	1.16 (1.11–1.22)	<.001
9–14 y	1		1	
Total No. of hospitalized episodes of fever	1.71 (1.60–1.84)	<.001	1.17 (1.08–1.26)	<.001
Severity of illness				
High temperature (>38°C)	1.95 (1.89–2.01)	<.001	1.35 (1.30–1.40)	<.001
Duration of fever	1.98 (1.95–2.00)	<.001	2.01 (1.98–2.04)	<.001
Hospitalized episode	9.21 (7.67–11.05)	<.001	3.61 (2.86–4.55)	<.001

Abbreviation: CI, confidence interval; INR, Indian rupees; OR, odds ratio.

In comparison, the sex of the child played an important role, with female children at a lower chance of receiving an antibiotic, after adjusting for all other risk factors (OR, 0.94 [95% CI, .90–.97]). Furthermore, children from a nuclear family also had a reduced chance of receiving an antibiotic for fever (OR, 0.90 [95% CI, .86–.94]) independent of the effect of the site, individual, and other social risk factors.

The antibiotic use at Pune was significantly higher than the other sites. This rural population was more affluent compared with the other sites that were predominantly in urban settlements (mean asset score: 11.5 vs 9.2, *P* < .001; mean monthly income [Indian rupee]: 20 663 vs 11 268, *P* < .001). They also had greater access to internet and motorized transportation (internet availability: 92.6% vs 77.7%, *P* < .001; motor transportation: 80.1% vs 46.3%, *P* < .001, respectively, between Pune and rest of the sites). Both the duration of fever (2.3 vs 2.5 days, *P* < .001) and highest temperature documented (37.4°C vs 37.8°C, *P* < .001) were lower at Pune than at the other sites.

## Discussion

Antibiotic use in febrile illness is commonplace in these cohorts, with children receiving 57.9 (95% CI, 57.2–58.6) fever-related antibiotic courses per 100 CYO. More than half of the children (55.9%) had at least 1 fever-related antibiotic course during the study. While other studies have extrapolated antibiotic data from Demographic and Health Surveys [5] or examined antibiotic prescriptions in younger cohorts [11], this study documented prospectively, using standardized tools and common protocols, antibiotic use in febrile illness in children of a wider pediatric age group from 4 geographically distinct parts of India.

Of those who received antibiotics, 64% had antibiotics initiated before the third day of fever. Very early initiation may reflect the lack of diagnostic tests and the demand from families paying out of pocket for healthcare for prompt resolution of symptoms. Based on the provisional diagnosis assigned by the treating physician, we note that the antibiotic usage was unlikely to have been warranted in 65.4% of the episodes where antibiotics were initiated. Fink et al, from a study on 8 LMICs, predominantly in Africa, reported unnecessary antibiotic usage rates ranging between 49% and 81% [5]. Our study documented that 24.3% episodes of antibiotics were discontinued within 3 days of initiation, suggesting that many of these episodes might have been self-limiting viral illnesses. On the other hand, early antibiotics could also prevent a more extensive bacterial illness and therefore may be lifesaving in resource-poor settings with inadequate health access.

Our study reports the widespread use of broad-spectrum antibiotics like amoxicillin and azithromycin for treating febrile illnesses. This aligns with observations in the World Health Organization report on surveillance of antibiotic consumption that noted that amoxicillin and azithromycin were the most commonly used antibiotics in most regions [12]. Because of the low cost and ease of administration, broad-spectrum antibiotics remain the preferred choice in resource-limited settings. The widespread use of azithromycin might result in antibiotic pressure leading to the emergence of resistance, as was observed in Pakistan and more recently in some parts of India [13,14].

We observed a higher risk of antibiotics among those with temperatures >38.4°C, which parents and local physicians consider a marker of severity of illness. Younger children, households with higher education, internet literacy, and the availability of personal conveyance were independently associated with a higher risk of antibiotic usage. Similarly, male children and those belonging to joint families were more likely to be initiated on antibiotics during a fever episode. Thus, both deep-rooted sociocultural and economic factors contribute to the prevailing antibiotic use practices. Though the systematic review by McKay et al on the factors associated with antibiotic usage among respiratory tract infections identified that clinical diagnosis and evidence from clinical examination were the principal drivers of antibiotic prescription [15], in the absence of low-cost point-of-care diagnostic tests, general practitioners are often compelled to initiate antibiotics at an early stage of the illness [15, 16]. We classified fever with rash and pharyngitis as possible bacterial etiology, as exclusion of bacterial etiology is challenging without investigations, in these settings where the burden of rheumatic heart disease has been a significant problem in the past.

The shorter duration of fever and lower temperature in febrile episodes noted in Pune might partly be explained by early and more aggressive management of febrile illness at this location.

This study has several limitations. We estimated incidence in children >6 months of age and do not provide information the first 6 months, when antibiotic use is likely to be relatively high [17, 18]. The frequency of illness and consequent hospitalizations in this age group drive the higher antibiotic use in these young infants [19]. Our estimates are only for fever-related antibiotic use, although children frequently receive antibiotics for respiratory and gastrointestinal ailments, as we and others have reported [11, 19]. Therefore, our estimates reflect only a subset of all antibiotic use in the population. This analysis was nested within a study aimed at estimating the incidence of typhoid fever. While no deliberate effort was made to influence antibiotic use, it is likely that offering blood cultures on the fourth day may have delayed antibiotics till then and increased the probability of receiving one after blood culture. The classification of fever episodes based on the diagnosis was arbitrary. We did not have access to other investigations, such as C-reactive protein or complete blood count, that could have helped make a more definitive classification. This is a limitation of the study.

The high incidence of antibiotic use and the inappropriate use of antibiotics pose a significant risk and can contribute to worsening antibiotic resistance in India. Both education at all levels of healthcare and communication to the public regarding inappropriate antibiotic use are needed to complement cost-effective diagnostic tests for an early identification of fever etiology in order to reduce inappropriate antibiotic usage.

## Supplementary Data

Supplementary materials are available at *The Journal of Infectious Diseases* online. Consisting of data provided by the authors to benefit the reader, the posted materials are not copyedited and are the sole responsibility of the authors, so questions or comments should be addressed to the corresponding author.

jiab115_suppl_Supplementary_Figure_1

jiab115_suppl_Supplementary_Tables_1-3

## Notes

***SEFI Consortium.*** Sites: Christian Medical College, Vellore (Anita Shirley David, Atrayee Nag, Malathi Murugesan, Nirmal Kumar, Prabakhar D. Moses, Ranjith Kumar R., Swathi Vajja); KEM Hospital and Research Centre, Pune (Bharat Choudhari, Sandeep Bhujbal, Sanjay Juvekar, Sonali Sanghavi, Tathagata Bhattacharjee); ICMR, National Institute of Cholera & Enteric Diseases, Kolkata (Debottam Pal, Jayanta Saha, Meghna Bannerjee, Nandini Chakrabarty, Pranab Chatterjee, Tania Pan); Centre for Health Research and Development–Society for Applied Studies, New Delhi (Alok Arya, Ananya Tupaki-Sreepurna, Ankita Dutta, Deepak More, Mohammed Aslam, Nidhi Goyal); Hamdard Institute of Medical Sciences and Research, New Delhi (Aparna Chakravarty, Chandra Mohan Kumar); central coordination team (Agila K. Pragasam, Dilesh Kumar A., Ira Praharaj, Jobin J. John, Nayana P. Nair, Nikhil Sahai, Priscilla Rupali, and Reshma Raju).

***Author contributions.*** J. J. and G. K. conceived and designed the study. J. J., G. K., A. B., T. R.-C., S. K., B. S., K. N. S., M. S., A. S. K., and P. S. developed the study protocol and analysis plan. J. J. and G. K. coordinated the project. M. S., K. N. S., A. B., A. S., T. R.-C., B. S., S. K., S. D., S. K. N., and B. V. coordinated data collection at the sites. A. S. K., K. R., J. J., and P. S. analyzed the data. A. S. K., M. S., and J. J. drafted the manuscript. All authors have reviewed and approved the final version of the manuscript. J. J. and G. K. had complete access to data and guarantee the manuscript.

***Acknowledgments.*** We thank Duncan Steele, Megan Carey, Supriya Kumar, Anita Zaidi, and the Bill & Melinda Gates Foundation for their technical support and review of the manuscript.

We also thank Nicholas Grassly (Imperial College London), Peter Smith (London School of Hygiene and Tropical Medicine), and Jayaprakash Muliyil (Christian Medical College Vellore), for assistance with study design and research proposal development. We thank the parents and children of the Surveillance for Enteric Fever in India (SEFI) study, and the SEFI field teams at the 4 sites that were involved in the sample and data collection.

***Financial support.*** This work was supported by Bill & Melinda Gates Foundation (grant number INV -009497-OPP1159351) and Fogarty International Center, National Institutes of Health, USA through D43 TW007392.

***Supplement sponsorship.*** This supplement is sponsored by the Christian Medical College Vellore Association.

***Potential conflicts of interest.*** All authors: No reported conflicts of interest.
