# Supplementary figures and images for: Antibiotics for Fever Among Children: Findings From the Surveillance for Enteric Fever in India Cohorts
Source: J Infect Dis. 2021 Nov 23;224(Suppl 5):S494–501. doi: 10.1093/infdis/jiab115 (PMC8892537; doi:10.1093/infdis/jiab115)

# Time to initiation of antibiotics for acute febrile illness among four pediatric cohorts (n=76027)

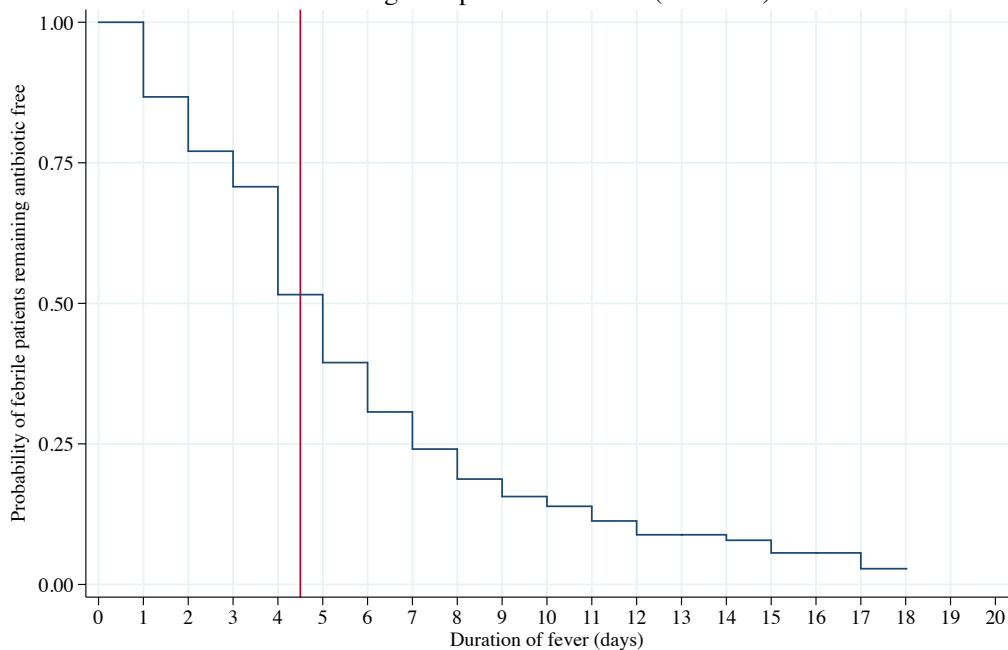

Supplement: jiab115_suppl_Supplementary_Figure_1 [file jiab115_suppl_supplementary_figure_1.pdf]
