# Supplementary material for: Antibiotics for Fever Among Children: Findings From the Surveillance for Enteric Fever in India Cohorts
Source: J Infect Dis. 2021 Nov 23;224(Suppl 5):S494–501. doi: 10.1093/infdis/jiab115 (PMC8892537; doi:10.1093/infdis/jiab115)
Supplement: jiab115_suppl_Supplementary_Tables_1-3 [file jiab115_suppl_supplementary_tables_1-3.docx]

## Supplementary Tables & Figures

**Supplementary Figure 1: Probability of febrile patients remaining antibiotic free among paediatric cohorts established as a part of the Surveillance for Enteric Fever in India (SEFI) network**

**Supplementary Table 1: Summary of the diagnosis of febrile episodes with at least three consecutive febrile days among the four paediatric cohorts established as part of the surveillance for enteric fever in India (SEFI) network**

| **Diagnosis** | **Initial diagnosis**  **(n= 16 200)** | **Final diagnosis**  **(n= 17 617)** |
| --- | --- | --- |
|  | **n (%)** | **n (%)** |
| **Probable bacterial aetiology** |  |  |
| Appendicitis | 1 (0.01%) | 3 (0.02%) |
| Ear infection | 61 (0.38%) | 61 (0.35%) |
| Enteric infection | 1 671 (10.31%) | 363 (2.06%) |
| LRTI | 2 129 (13.14%) | 1 986 (11.27%) |
| Scrub Typhus |  | 3 (0.02%) |
| Skin & soft tissue infection | 61 (0.38%) | 48 (0.27%) |
| Tuberculosis | 2 (0.01%) | 5 (0.03%) |
| Urinary tract infection | 129 (0.80%) | 84 (0.48%) |
| Other confirmed bacterial infections | 15 (0.09%) | 34 (0.19%) |
| **Possible bacterial aetiology** |  |  |
| Acute gastroenteritis | 568 (3.51%) | 497 (2.82%) |
| Eye infections | 15 (0.09%) | 23 (0.13%) |
| Fever with rash | 20 (0.12%) | 85 (0.48%) |
| Pharyngitis | 177 (1.09%) | 161 (0.91%) |
| Tonsillitis | 248 (1.53%) | 224 (1.27%) |
| Other illness | 41 (0.25%) | 65 (0.37%) |
| **Unlikely bacterial aetiology** |  |  |
| Acute undifferentiated febrile illness | 5 736 (35.41%) | 8 167 (46.36%) |
| Dengue fever | 25(0.15%) | 139 (0.79%) |
| Jaundice | 33 (0.20%) | 53 (0.30%) |
| Measles | 8 (0.05%) | 9 (0.05%) |
| Mumps | 62 (0.38%) | 69 (0.39%) |
| URTI | 5 121 (31.61%) | 5 454 (30.96%) |
| Varicella | 77 (0.48%) | 84 (0.48%) |

**Supplementary Table 2: Total child-years of observation (CYO), incidence of antibiotic, and multiple antibiotic usage for febrile illness among the four paediatric cohorts established as part of the surveillance for enteric fever in India (SEFI) network**

| **Age group** | **Child-years**  **of observation** | **Antibiotic episodes**  **per 100CYO** | **Multiple antibiotic**  **episodes per 100CYO** |
| --- | --- | --- | --- |
| **Delhi** |  |  |  |
| 6 m - 4 years | 2 831.7 | 81.4 (78.1 - 84.8) | 14.4 (13.1 - 16.0) |
| 5 - 9 years | 4 693.5 | 41.4 (39.6 - 43.3) | 7.9 (7.1 - 8.7) |
| 10 - 14 years | 3 754.8 | 27.2 (25.6 - 29.0) | 5.4 (4.7 - 6.2) |
| Overall | 11 280.0 | 46.7 (45.5 - 48.0) | 8.7 (8.2 - 9.3) |
| **Kolkata** |  |  |  |
| 6 m - 4 years | 3 019.1 | 71.4 (68.4 - 74.5) | 11.3 (10.1 - 12.5) |
| 5 - 9 years | 4 070.5 | 41.2 (39.2 - 43.2) | 6.0 (5.3 - 6.8) |
| 10 - 14 years | 4 539.5 | 29.3 (27.8 - 31.0) | 4.3 (3.7 - 4.9) |
| Overall | 11 629.5 | 44.4 (43.2 - 45.6) | 6.7 (6.2 - 7.2) |
| **Vellore** |  |  |  |
| 6 m - 4 years | 3 081.4 | 87.8 (84.5 - 91.2) | 11.4 (10.2 - 12.6) |
| 5 - 9 years | 5 072.1 | 46.4 (44.5 - 48.3) | 6.1 (5.4 - 6.8) |
| 10 - 14 years | 4 378.7 | 30.1 (28.5 - 31.8) | 4.4 (3.8 - 5.0) |
| Overall | 12 532.2 | 50.9 (49.6 - 52.1) | 6.8 (6.3 – 7.3) |
| **Pune** |  |  |  |
| 6 m - 4 years | 2 812.8 | 142.5 (138.2 - 147.0) | 23.9 (22.2 - 25.8) |
| 5 - 9 years | 4 223.7 | 92.6 (89.7 - 95.5) | 14.6 (13.4 - 15.8) |
| 10 - 14 years | 4 481.1 | 54.8 (52.6 - 57.0) | 7.0 (6.2 - 7.8) |
| Overall | 11 517.6 | 90.1 (88.3 - 91.8) | 13.9 (13.2 - 14.6) |
| **All four sites** |  |  |  |
| 6 m - 4 years | 11 745.1 | 95.1 (93.4 - 96.9) | 15.1 (14.4 - 15.8) |
| 5 - 9 years | 18 059.8 | 54.7 (53.6 - 55.8) | 8.5 (8.1 - 9.0) |
| 10 - 14 years | 17 154.4 | 35.7 (34.8 - 36.6) | 5.2 (4.9 - 5.6) |
| Overall | 46 959.3 | 57.9 (57.2 - 58.6) | 9.0 (8.7 - 9.2) |

**Supplementary Table 3: Incidence of individual antibiotic usage for febrile illness among the four paediatric cohorts established as part of the surveillance for enteric fever in India (SEFI) network**

| **Age group** | **Azithromycin**  **per 100CYO** | **Amoxicillin**  **per 100CYO** | **Cephalosporin**  **per 100CYO** |
| --- | --- | --- | --- |
| **Delhi** |  |  |  |
| 6 m - 4 years | 16.6 (15.1 - 18.1) | 44.3 (41.9 - 46.8) | 20.7 (19.1 - 22.5) |
| 5 - 9 years | 12.4 (11.4 - 13.4) | 20.7 (19.4 - 22.0) | 11.4 (10.5 - 12.4) |
| 10 - 14 years | 11.6 (10.6 - 12.8) | 10.5 (9.5 - 11.6) | 7.4 (6.5 - 8.3) |
| Overall | 13.2 (12.5 - 13.9) | 23.2 (22.3 - 24.1) | 12.4 (11.8 - 13.1) |
| **Kolkata** |  |  |  |
| 6 m - 4 years | 8.9 (7.9 - 10.1) | 24.1 (22.4 – 26.0) | 30.9 (28.9 - 32.9) |
| 5 - 9 years | 8.5 (7.7 - 9.5) | 10.0 (9.1 - 11.0) | 18.2 (16.9 - 20.0) |
| 10 - 14 years | 8.2 (7.4 - 9.1) | 7.0 (6.2 - 7.8) | 11.0 (10.0 - 12.0) |
| Overall | 8.5 (8.0 - 9.1) | 12.5 (11.9 - 13.2) | 18.7 (17.9 - 19.5) |
| **Vellore** |  |  |  |
| 6 m - 4 years | 40.4 (38.2 - 42.7) | 25.6 (23.8 - 27.5) | 15.9 (14.5 - 17.4) |
| 5 - 9 years | 23.5 (22.2 - 24.9) | 12.9 (11.9 - 13.9) | 7.1 (6.4 - 7.8) |
| 10 - 14 years | 14.5 (13.4 - 15.6) | 7.8 (7.0 - 8.7) | 6.3 (5.6 - 7.1) |
| Overall | 24.5 (23.6 - 25.4) | 14.2 (13.6 - 14.9) | 9.0 (8.5 - 9.5) |
| **Pune** |  |  |  |
| 6 m - 4 years | 20.4 (18.7 - 22.1) | 90.2 (86.7 - 93.8) | 15.0 (13.6 - 16.5) |
| 5 - 9 years | 11.4 (10.4 - 12.5) | 54.4 (52.2 - 56.7) | 14.0 (12.9 - 15.2) |
| 10 - 14 years | 7.6 (6.8 - 8.4) | 24.0 (22.6 - 25.5) | 12.1 (11.1 - 13.2) |
| Overall | 12.1 (11.5 - 12.8) | 51.3 (50.0 - 52.6) | 13.5 (12.9 - 14.2) |
| **All four sites** |  |  |  |
| 6 m - 4 years | 21.8 (20.9 - 22.6) | 45.2 (44.0 - 46.4) | 20.7 (19.9 - 21.5) |
| 5 - 9 years | 14.4 (13.8 - 15.0) | 24.0 (23.3 - 24.7) | 12.3 (11.8 - 12.9) |
| 10 - 14 years | 10.4 (9.9 - 10.9) | 12.4 (11.9 - 13.0) | 9.3 (8.8 - 9.8) |
| Overall | 14.8 (14.4 - 15.1) | 25.1 (24.6 - 25.5) | 13.3 (13.0 - 13.7) |

**Supplementary Table 4: Generalised estimation equation (GEE) model for the risk of antibiotic usage for febrile illness per individual sites established as part of the surveillance for enteric fever in India (SEFI) network**

| **Variables** | **Unadjusted** | | **Adjusted** | |
| --- | --- | --- | --- | --- |
|  | **OR (95% CI)** | **p-value** | **OR (95% CI)** | **p-value** |
| **DELHI** |  |  |  |  |
| **Social factors** |  |  |  |  |
| Post secondary education | 1.06 (0.98 -1.14) | 0.144 | 1.06 (0.97 - 1.16) | 0.226 |
| Internet accessibility | 1.07 (0.95 -1.19) | 0.234 | 1.10 (0.97-1.26) | 0.149 |
| Conveyance (motor vehicle) | 1.03 (0.95 -1.11) | 0.453 | 0.98 (0.89-1.07) | 0.627 |
| Nuclear family | 0.80 (0.74-0.87) | <0.001 | 0.88 (0.80-0.97) | 0.013 |
| Monthly income>10000 INR |  |  | 1.03 (0.94-1.13) | 0.479 |
| **Individual factors** |  |  |  |  |
| Female gender | 0.95 (0.88-1.03) | 0.212 | 0.90 (0.83-0.98) | 0.019 |
| Age category |  |  |  |  |
| 6 m - 4 years | 1.59 (1.44-1.75) | <0.001 | 1.80 (1.61-2.01) | <0.001 |
| 5 - 9 years | 1.30 (1.18-1.43) | <0.001 | 1.39 (1.25-1.55) | <0.001 |
| 9 -14 years | 1 |  | 1 |  |
| Total number of hospitalised episodes of fever | 1.74 (1.45-2.09) | <0.001 | 1.23 (0.99-1.53) | 0.065 |
| **Severity of illness** |  |  |  |  |
| High Temperature (>38°C) | 0.58 (0.54- 0.62) | <0.001 | 0.51 (0.48- 0.55) | <0.001 |
| Duration of fever | 1.69 (1.65-1.73) | <0.001 | 1.71 (1.67-1.75) | <0.001 |
| Hospitalized episode | 4.23 (2.76-6.49) | <0.001 | 1.60 (0.90-2.84) | 0.109 |
| **KOLKATA** |  |  |  |  |
| **Social factors** |  |  |  |  |
| Post secondary education | 0.92 (0.84-1.01) | 0.080 | 0.97 (0.86-1.09) | 0.569 |
| Internet accessibility | 0.89 (0.82-0.97) | 0.009 | 0.98 (0.88-1.09) | 0.766 |
| Conveyance (motor vehicle) | 0.86 (0.78-0.94) | 0.001 | 0.80 (0.71-0.91) | 0.001 |
| Nuclear family | 0.75 (0.70-0.81) | <0.001 | 0.78 (0.71-0.85) | <0.001 |
| Monthly income>10000 INR |  |  | 1.09 (0.97-1.21) | 0.139 |
| **Individual factors** |  |  |  |  |
| Female gender | 1.02 (0.95-1.10) | 0.623 | 1.01 (0.92-1.10) | 0.846 |
| Age category |  |  |  |  |
| 6 m - 4 years | 1.65 (1.51-1.81) | <0.001 | 1.47 (1.32-1.64) | <0.001 |
| 5 - 9 years | 1.18 (1.08-1.29) | <0.001 | 1.06 (0.95-1.19) | 0.261 |
| 9 -14 years | 1 |  | 1 |  |
| Total number of hospitalised episodes of fever | 1.70 (1.36-2.11) | <0.001 | 1.21 (0.89-1.64) | 0.222 |
| **Severity of illness** |  |  |  |  |
| High Temperature (>38°C) | 3.82 (3.54-4.13) | <0.001 | 1.73 (1.58-1.89) | <0.001 |
| Duration of fever | 2.78 (2.69-2.88) | <0.001 | 2.61 (2.52-2.70) | <0.001 |
| Hospitalized episode | 4.43 (2.89-6.78) | <0.001 | 0.82 (0.43-1.57) | 0.556 |

**Supplementary Table 4: Generalised estimation equation (GEE) model for the risk of antibiotic usage for febrile illness per individual sites in India established as part of the surveillance for enteric fever in India (SEFI) network [contd.]**

| **Variables** | **Unadjusted** | | **Adjusted** | |
| --- | --- | --- | --- | --- |
|  | **OR (95% CI)** | **p-value** | **OR (95% CI)** | **p-value** |
| **VELLORE** |  |  |  |  |
| **Social factors** |  |  |  |  |
| Post secondary education | 1.16 (1.09-1.24) | <0.001 | 1.10 (1.00-1.19) | 0.038 |
| Internet accessibility | 1.22 (1.14-1.31) | <0.001 | 1.10 (0.99-1.20) | 0.076 |
| Conveyance (motor vehicle) | 1.20 (1.12-1.29) | <0.001 | 1.22 (1.11-1.34) | <0.001 |
| Nuclear family | 0.88 (0.83-0.94) | <0.001 | 0.96 (0.88-1.04) | 0.302 |
| Monthly income>10000 INR |  |  | 1.07 (0.97-1.18) | 0.173 |
| **Individual factors** |  |  |  |  |
| Female gender | 0.94 (0.88-1.00) | 0.055 | 0.97 (0.90-1.05) | 0.443 |
| Age category |  |  |  |  |
| 6 m - 4 years | 1.19 (1.10-1.29) | <0.001 | 0.98 (0.88-1.08) | 0.637 |
| 5 - 9 years | 1.12 (1.03-1.21) | 0.007 | 0.98 (0.88-1.08) | 0.639 |
| 9 -14 years | 1 |  | 1 |  |
| Total number of hospitalised episodes of fever | 1.60 (1.47-1.74) | <0.001 | 1.10 (0.97-1.24) | 0.139 |
| **Severity of illness** |  |  |  |  |
| High Temperature (>38°C) | 3.93 (3.70-4.19) | <0.001 | 1.74 (1.61-1.87) | <0.001 |
| Duration of fever | 2.30 (2.25-2.36) | <0.001 | 2.17 (2.12-2.22) | <0.001 |
| Hospitalized episode | 15.10 (11.09-20.55) | <0.001 | 6.60 (4.44-9.83) | <0.001 |
| **PUNE** |  |  |  |  |
| **Social factors** |  |  |  |  |
| Post secondary education | 1.23 (1.14-1.31) | <0.001 | 1.12 (1.03-1.21) | 0.006 |
| Internet accessibility | 1.34 (1.18-1.52) | <0.001 | 1.22 (1.06-1.41) | 0.006 |
| Conveyance (motor vehicle) | 1.36 (1.25-1.47) | <0.001 | 1.31 (1.19-1.44) | <0.001 |
| Nuclear family | 0.88 (0.82-0.95) | 0.001 | 0.98 (0.90-1.07) | 0.668 |
| Monthly income>10000 INR |  |  | 1.09 (1.00-1.19) | 0.059 |
| **Individual factors** |  |  |  |  |
| Female gender | 0.89 (0.84-0.96) | 0.001 | 0.87 (0.81-0.94) | <0.001 |
| Age category |  |  |  |  |
| 6 m - 4 years | 1.32 (1.22-1.44) | <0.001 | 1.28 (1.17-1.40) | <0.001 |
| 5 - 9 years | 1.28 (1.19-1.39) | <0.001 | 1.21 (1.11-1.32) | <0.001 |
| 9 -14 years | 1 |  | 1 |  |
| Total number of hospitalised episodes of fever | 1.72 (1.51-1.95) | <0.001 | 1.19 (1.03-1.37) | 0.017 |
| **Severity of illness** |  |  |  |  |
| High Temperature (>38°C) | 2.28 (2.15-2.41) | <0.001 | 1.64 (1.54-1.74) | <0.001 |
| Duration of fever | 1.78 (1.74-1.83) | <0.001 | 1.69 (1.64-1.74) | <0.001 |
| Hospitalized episode | 14.18 (9.05-22.24) | <0.001 | 6.34 (3.89-10.33) | <0.001 |
